# A Detailed Review of Surgical Management of Uncommon Cutaneous Disorders

**DOI:** 10.7759/cureus.36763

**Published:** 2023-03-27

**Authors:** Aditya Tolat, Dheer S Kalwaniya, Ashok Sharma, Devender Kumar, Shivangi Rana, Charanjeet Ahluwalia

**Affiliations:** 1 General Surgery, Vardhman Mahavir Medical College and Safdarjung Hospital, New Delhi, IND; 2 Dermatology, NRI (Non-Resident Indian) Institute of Medical Sciences and ANH (Anil Neerukonda Hospital), Visakhapatnam, IND; 3 Pathology, Vardhman Mahavir Medical College and Safdarjung Hospital, New Delhi, IND

**Keywords:** dermatofibrosarcoma protuberans, skin disease/dermatology, malignant proliferating trichilemmal cyst, malignant chondroid syringoma, mycetoma foot, plexiform neurofibromas, surgery general

## Abstract

A variety of cutaneous disorders can present to the general surgeon either directly or by referral for surgical intervention. Some conditions are commonly seen and operated on by general surgeons which include lipoma, epidermoid cyst, etc. On the other hand, some are uncommon conditions like dermatofibrosarcoma protuberans and chondroid syringoma which require a high index of suspicion to diagnose. Most general surgeons are not familiar with the latest guidelines to treat such uncommon conditions. In this article, we provide a review of uncommon cutaneous disorders requiring surgical intervention that were encountered at our high-volume tertiary care center and a discussion about their etiology, presentation, diagnosis, management and follow-up with one case report of each condition. The objective of this article is to familiarize the general surgeon with these cutaneous disorders which though uncommon, will present in their practice at some point.

## Introduction

It is common in surgical practice for a patient to present with a swelling that may be clearly visible externally or a mass palpable just under the skin. Most of these swellings or masses are benign in nature and familiar to the surgeon. Such swellings or masses are easily excised after appropriate investigations. However, occasionally a patient presents to the surgeon with a swelling or mass, where the clinical picture does not fit that of the common conditions that are encountered in regular practice. Sometimes, a seemingly benign swelling or mass may actually be a malignant lesion. In this article, we describe such uncommon conditions that are presented to our high-volume tertiary care center in India. We discuss the incidence, presentation, examination, investigation and management of such patients along with clinical and histopathological images. We intend to familiarize the general surgeon with these conditions that they may encounter during their practice. In this article, we discuss the following conditions: dermatofibrosarcoma protuberans, plexiform neurofibroma, chondroid syringoma, trichilemmal cyst, mycetoma foot and button osteoma along with a case report for each condition.

## Materials and methods

The retrospective case series was compiled from the existing data from the Department of General Surgery, Vardhman Mahavir Medical College and Safdarjung Hospital, New Delhi. Data was collected retrospectively for the time period of November 2021 to November 2022. Patients with uncommon visible swellings or palpable masses, whose data was available from their first visit till three to five months in the postoperative period (depending on case to case), were included in this study. The detailed clinical examination findings and investigations were noted. Each patient's surgical procedure was noted, and the histopathological examination report of the excised specimens was traced. All the collected clinical data and histopathology findings were compared with the existing literature.

Dermatofibrosarcoma protuberans was initially excised without margins; however, following histopathological reporting, revision surgery was done and margins were revised to 4 cm in all directions including the deep fascia. Plexiform neurofibroma was excised with a margin of 1 cm sparing the deep fascia. Skin flaps on either side were mobilized to facilitate primary closure. Chondroid syringoma of the scalp was excised preserving as much normal tissue as possible. The defect was closed using a transposition flap from the adjacent scalp, and the resultant defect was covered with a split skin graft. Trichilemmal cysts were excised completely with their wall without any rupture and primary closure was done. Mycetoma of the foot was debrided radically sparing the tendons and bones, and the wound was allowed to heal by secondary intention. Button osteoma was excised using a chisel and mallet, and the wound closed primarily.

## Results

Case 1

**Figure 1:**
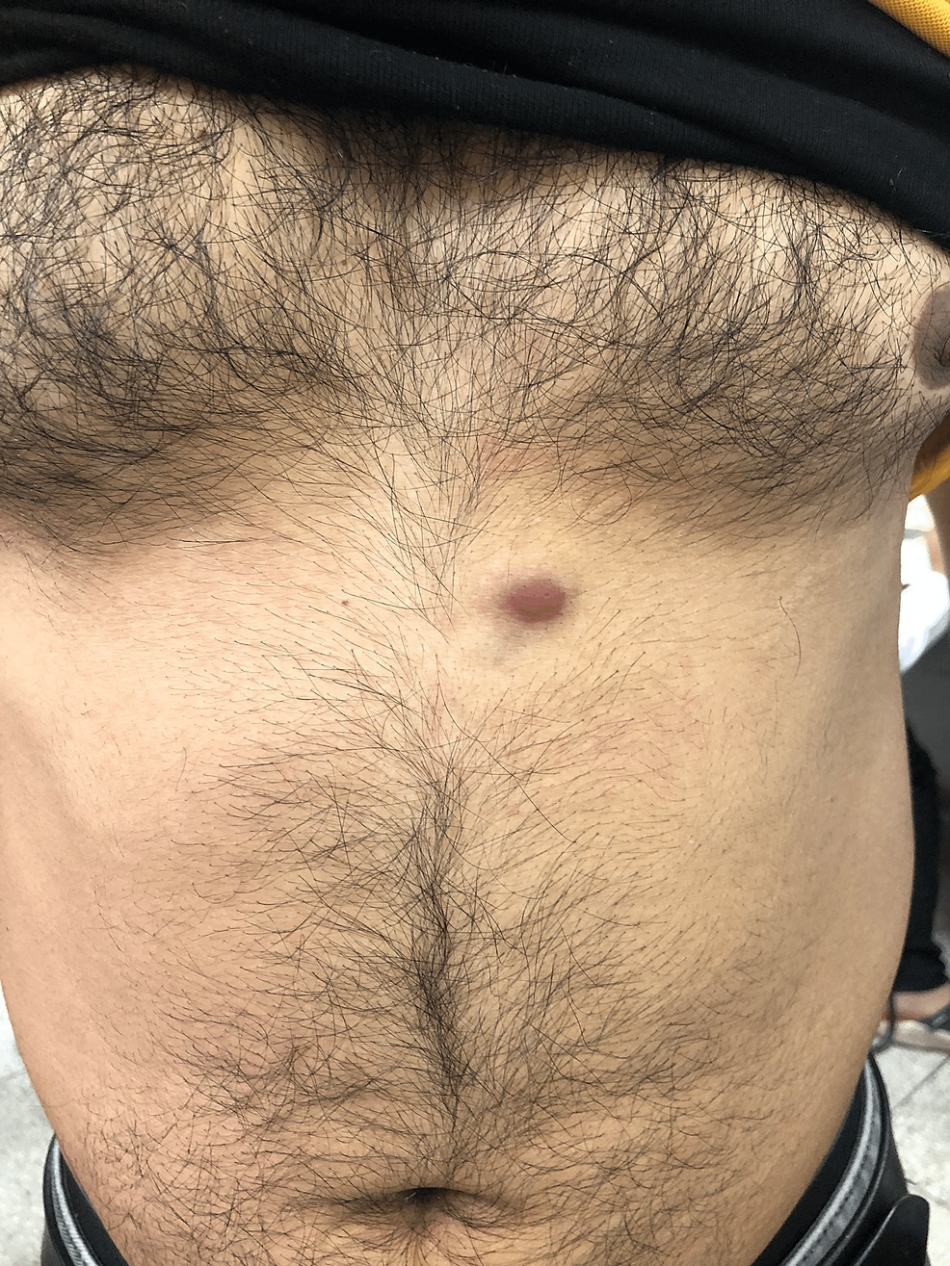
On presentation, visible mass in the left hypochondrium with purple discoloration of the overlying skin.

A 29-year-old male presented to the outpatient department with complaints of multiple masses (one large and others small) over the upper anterior abdominal wall and anterior wall of the lower thorax for two years. The patient had initially noticed the mass in the upper left quadrant of the abdomen two years ago when it was approximately 0.5x0.5cm. It was insidious in onset and slowly progressed in size to 1x1 cm over two years. It was painless and not associated with any discharge. One year ago, the patient had noticed multiple smaller masses of 0.5x0.5 cm in the region surrounding the primary mass and one mass of 0.5x0.5 cm over the lower anterior chest. There was no history suggestive of constitutional symptoms or distant metastasis. On examination, there was a mass of 1x1 cm in the left hypochondrium 3 cm lateral to midline with purple discoloration of the overlying skin. The overlying skin temperature was normal; the mass was non-tender, the surface was smooth, the margins were well-defined and the consistency was firm. It was non-compressible and non-reducible, and the cough impulse was absent. It was non-pulsatile. Mass was adherent to the skin and free from the underlying muscle. There were three masses of 0.5x0.5 cm each just superior and medial to the primary mass and another mass of 0.5x0.5 cm in the midline over the sternum 3 cm superior to the xiphoid (Figure 1).

**Figure 2:**
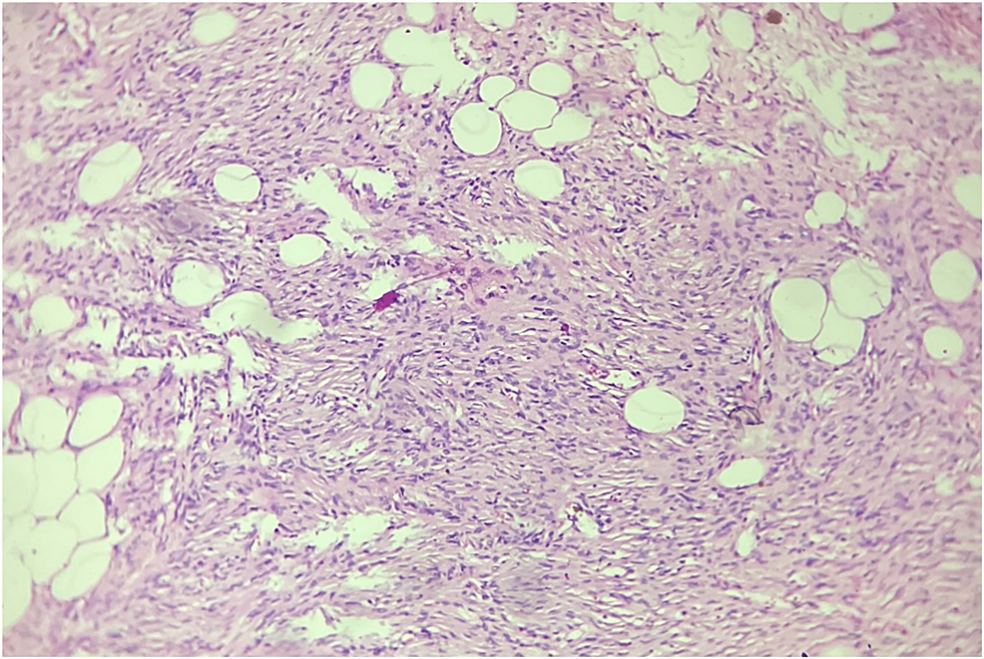
Storiform arrangement of spindle cells and entrapping subcutaneous fat in dermatofibrosarcoma protuberans, H&E 40X. H&E: hematoxylin and eosin stain, 40X: 40 times magnification.

**Figure 3:**
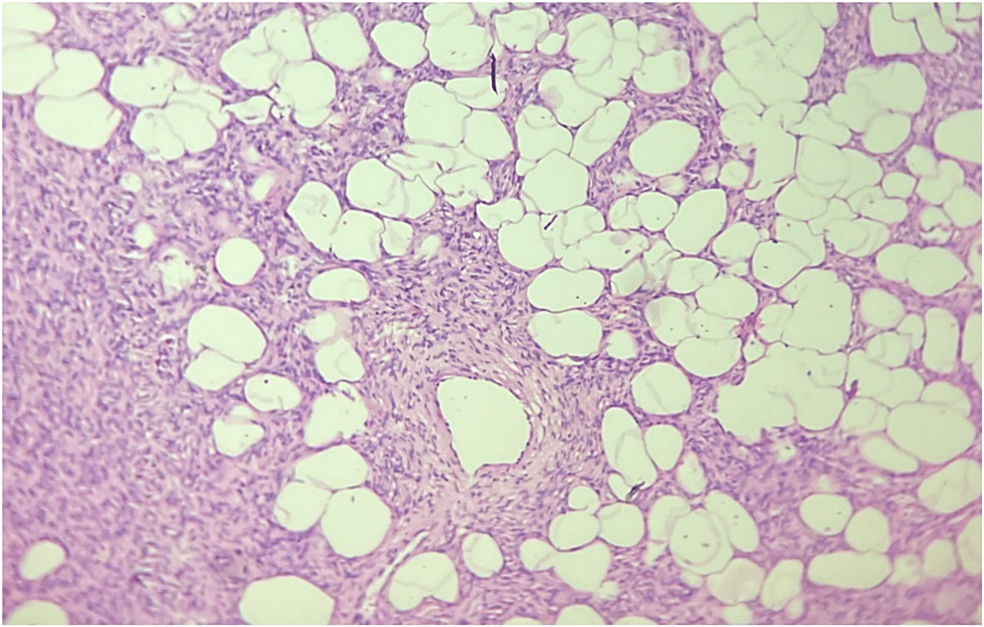
Spindle cells entrapping subcutaneous fat in dermatofibrosarcoma protuberans giving a honeycomb appearance, H&E 40X. H&E: hematoxylin and eosin stain, 40X: 40 times magnification.

**Figure 4:**
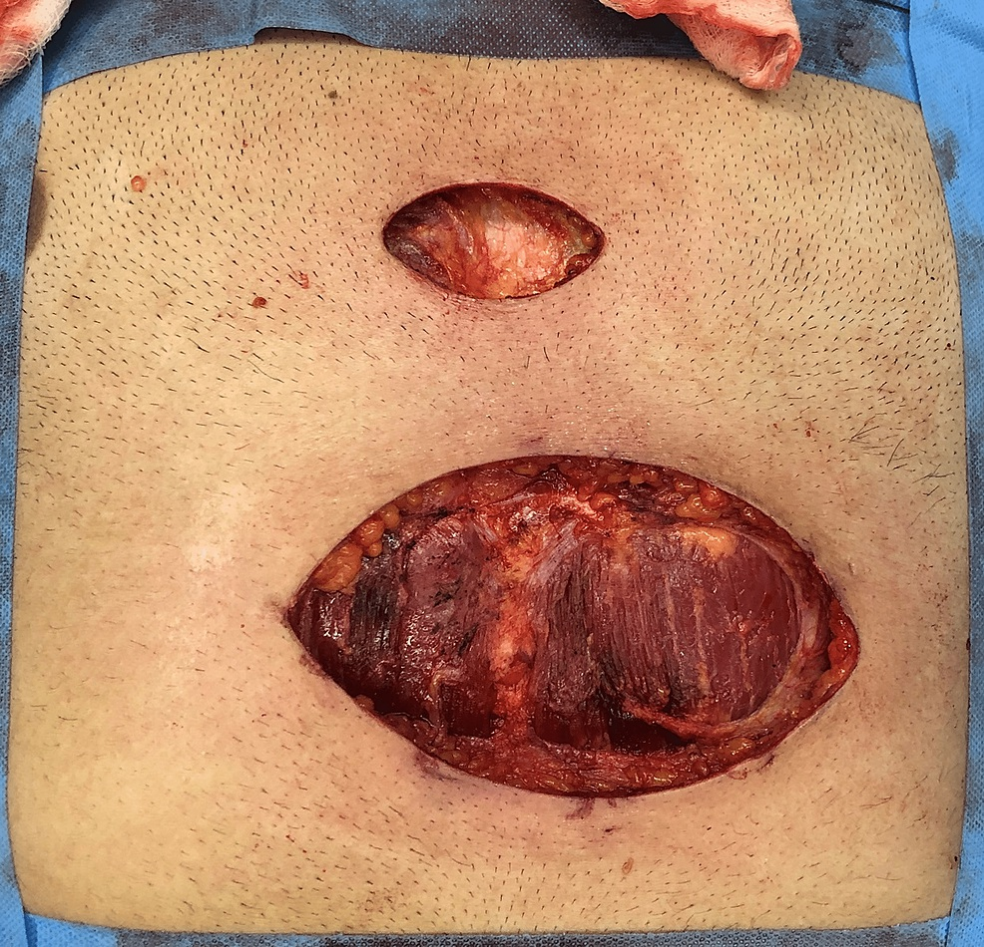
Revision surgery with wide margins.

The patient had already been investigated at another center with ultrasonography suggesting a subcutaneous mass. Fine-needle aspiration cytology (FNAC) showed fibrofatty fragments suggestive of lipoma. An excision biopsy was performed on the primary mass and surrounding three smaller masses which could be accessed from the incision. The mass on the sternum was not excised at this stage. Histopathology examination (HPE) report revealed a spindle cell neoplasm arranged in a storiform and whorling pattern. The cells were spindle-shaped with moderate eosinophilic cytoplasm, monomorphic nuclei and inconspicuous nucleoli. Mitotic activity was rare. A tumor was seen infiltrating the subcutaneous fat with a honeycomb pattern, and a deep-resected margin was involved by the tumor. On immunohistochemistry, the cells were positive for vimentin and CD34. Ki67 was 2% (Figures 2, 3). Thus, a diagnosis of dermatofibrosarcoma protuberans was made. The patient was counseled, and a revision wide local excision with margins of 4 cm in all directions and depth up to deep fascia was performed (Figure 4). The mass over the sternum was also excised with wide margins in a similar manner.

**Figure 5:**
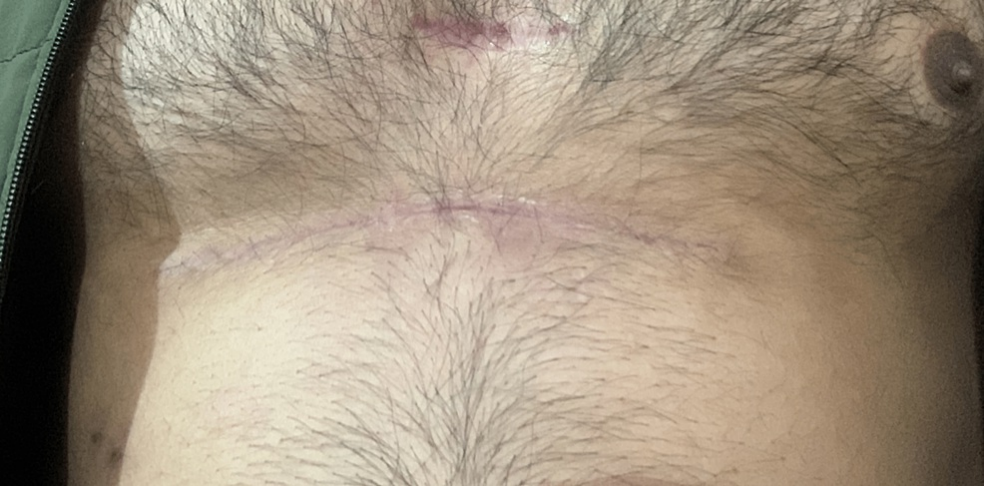
Three months post-surgery.

Subsequent HPE report revealed tumor-free margins. The patient followed up regularly to allow early detection of local recurrence if it occurs (Figure 5).

Case 2

**Figure 6:**
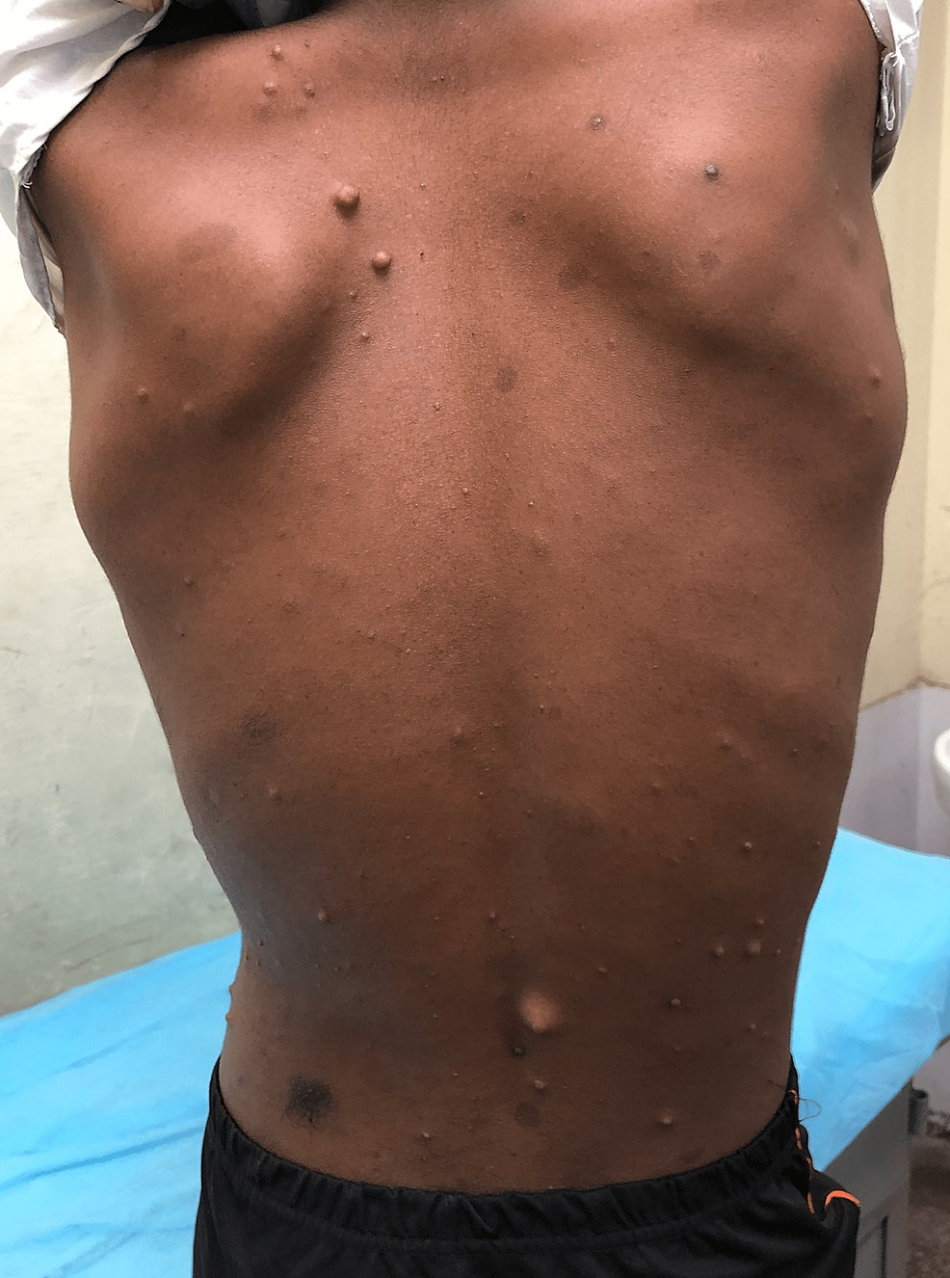
Multiple nodules and cafe au lait spots.

**Figure 7:**
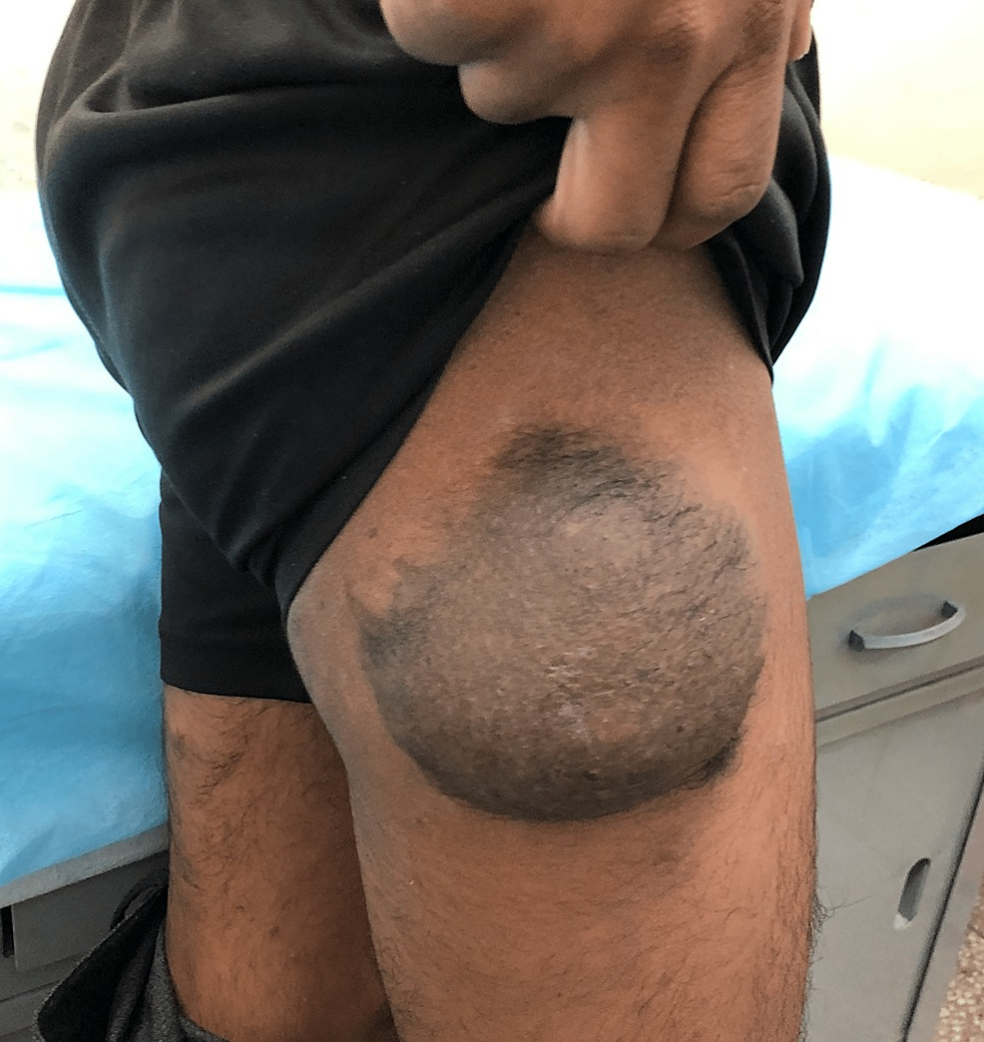
A 10x10 cm hyperpigmented lesion on the right anterolateral thigh.

A 16-year-old patient was referred to our center in view of a large swelling on the outer aspect of the right thigh for one and a half years. The patient had a history of multiple small nodules all over the body since childhood. The nodules had developed spontaneously and were painless, not increasing or decreasing in size, not regressing spontaneously. There was no history of auditory or visual complaints. There was no family history of neurofibromatosis. One and a half years back, the patient noticed a swelling on the right thigh outer aspect which was a little larger than the other nodules approximately 2x2 cm, and unlike other swellings, it was associated with blackish discoloration of the overlying skin. The swelling progressively increased in size to the current size of 10x10 cm. On examination, the patient was adequately built and moderately nourished. Multiple cafe au lait spots were noted over the trunk. There were multiple non-tender, firm, skin-colored nodules all over the body ranging from 0.5x0.5 cm to 1x1 cm. The nodules were compressible with a palpable defect in the fascia (Figure 6). On the lateral aspect of the right upper thigh, there was a large swelling of 10x10 cm. The swelling was spherical and non-pulsatile with overlying hyperpigmentation and excess hair. It was non-tender, with a normal overlying temperature. The surface was smooth, the edge was ill-defined and the consistency was soft and uniform. It was non-fluctuant and non-transilluminant. It was fixed to the overlying skin but free from underlying muscle (Figure 7).

**Figure 8:**
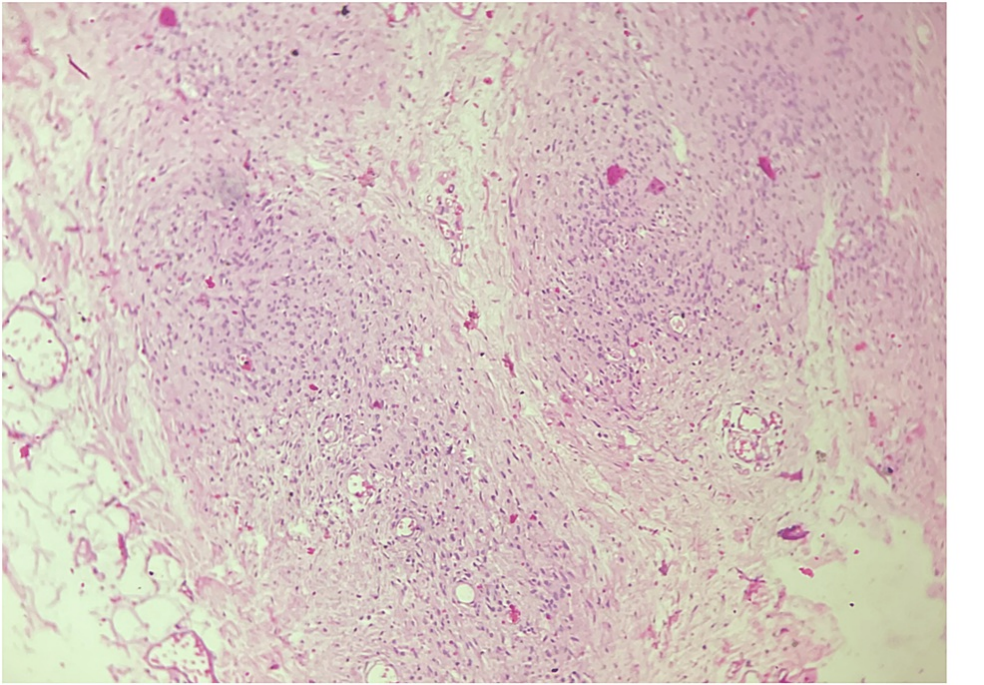
Irregularly expanded nerve bundles having Schwann cells with wire-like collagen in neurofibroma, H&E 10X. H&E: hematoxylin and eosin stain, 10X: 10 times magnification.

Based on all features, a diagnosis of sporadic neurofibromatosis-1 with plexiform neurofibroma of the right thigh was made. Ophthalmology and ENT consultations were obtained to screen for other associated abnormalities. Add-on contrast-enhanced computed tomography (CECT) of the abdomen and thorax revealed no abnormality. MRI of the right thigh revealed the swelling to be confined to subcutaneous tissue, and thus, the patient was posted for resection of the tumor with a 1 cm margin. Primary closure of the defect was achieved. Histopathology report confirmed the diagnosis of plexiform neurofibroma on account of irregularly expanded nerve bundles having Schwann cells with wire-like collagen in neurofibroma with S-100 positive (Figure 8).

**Figure 9:**
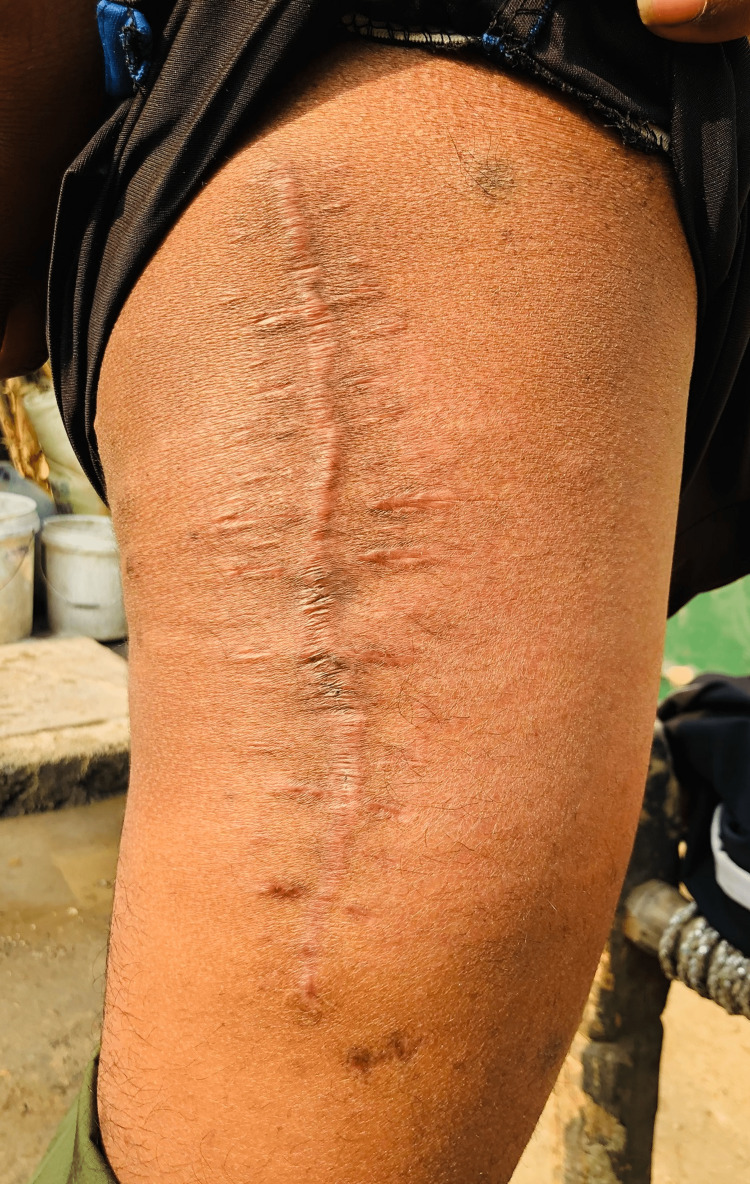
Four months post-excision.

The patient was followed up to look for recurrence or de novo plexiform neurofibroma (Figure 9).

Case 3

**Figure 10:**
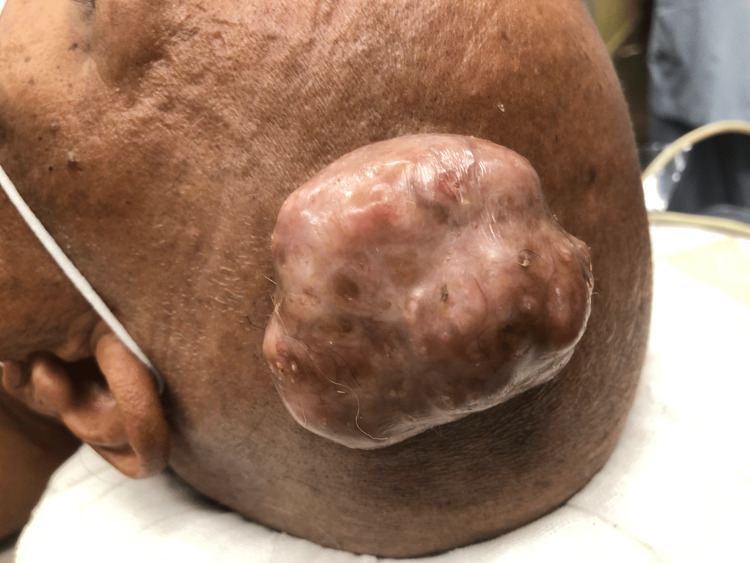
On presentation, 7x7 cm swelling on the left temporoparietal region.

A 53-year-old male presented to the outpatient department with a complaint of swelling on the left side of the head for four years. The patient was apparently asymptomatic four years ago when he noticed a swelling on the left side of the head above the ear. It was insidious at the onset of approximately 1x1 cm and gradually increased to the size of 7x7 cm. It was not associated with pain. There was no history of trauma or masses elsewhere in the body. There was no history suggestive of constitutional symptoms or distant metastasis. On examination, there was a 7x7 cm swelling on the scalp at the junction of the left frontal and parietal region. It was irregular in shape, skin-colored with an irregular surface and well-defined edge. It was non-pulsatile, and cough impulse was not present. The skin over the swelling was stretched with visible veins. The overlying temperature was normal, and the swelling was non-tender. The surface had a nodular feel, and the consistency was firm. It was non-compressible and non-reducible. It was fixed to the overlying skin but free from underlying bone (Figure 10). There was no cervical lymphadenopathy.

**Figure 11:**
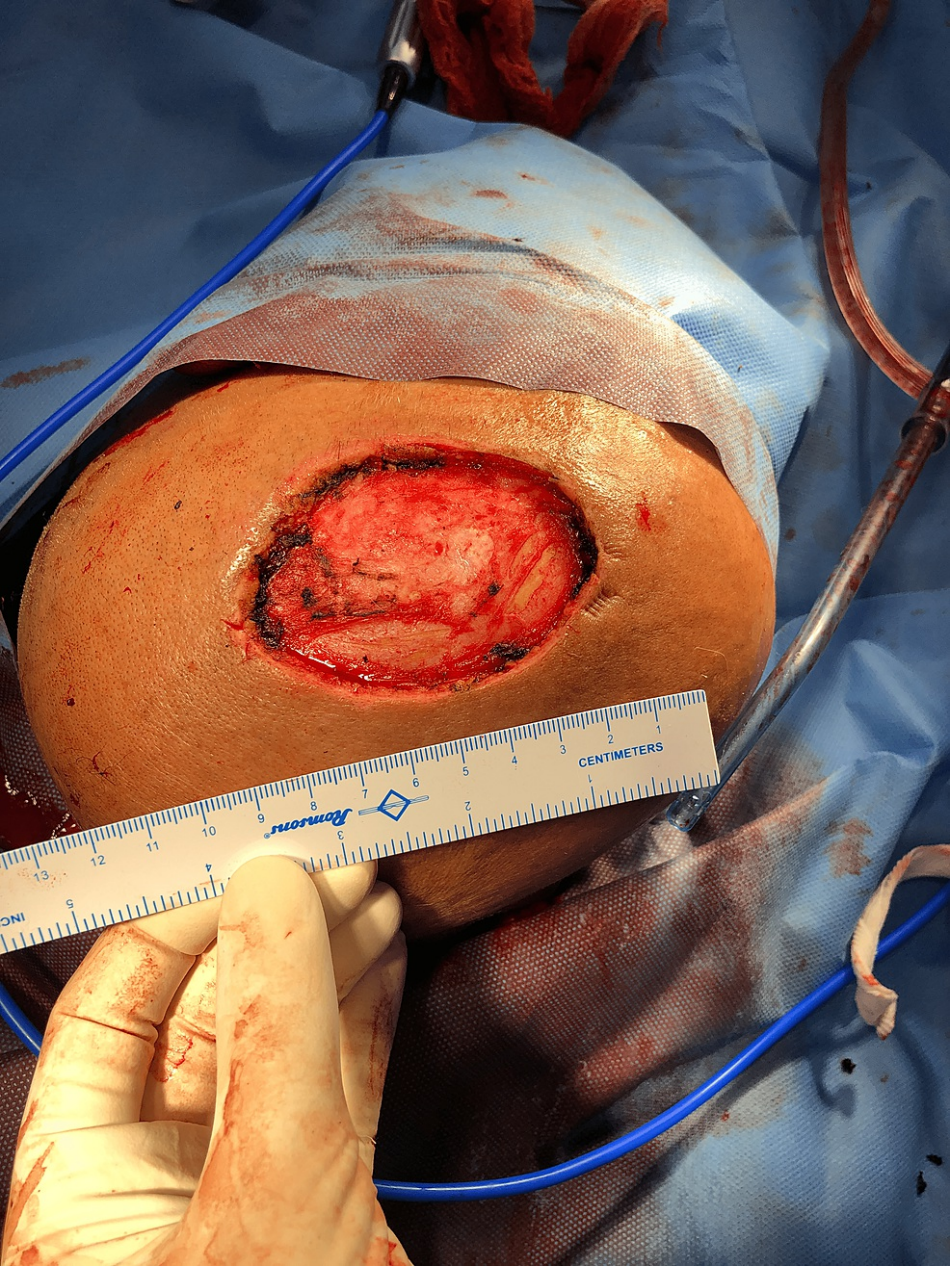
Post-excision defect.

Fine-needle aspiration cytology revealed epithelial and myoepithelial cells in a fibrillary and chondromyxoid stromal background suggestive of chondroid syringoma. Non-contrast CT scan of the head confirmed the swelling to be free from the calvarium. Excision of the tumor was done with grossly negative margins (Figure 11).

**Figure 12:**
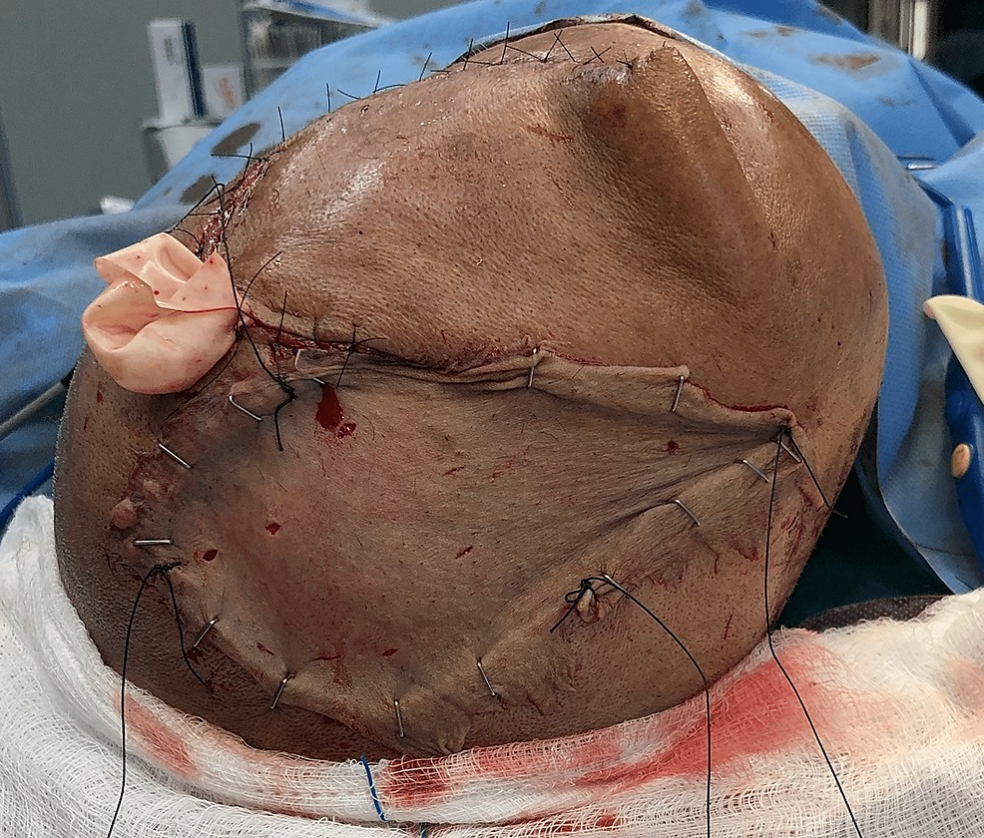
Reconstruction with transposition flap.

The resultant defect was closed with a transposition flap, and split skin grafting of the resultant scalp defect was done (Figure 12).

**Figure 13:**
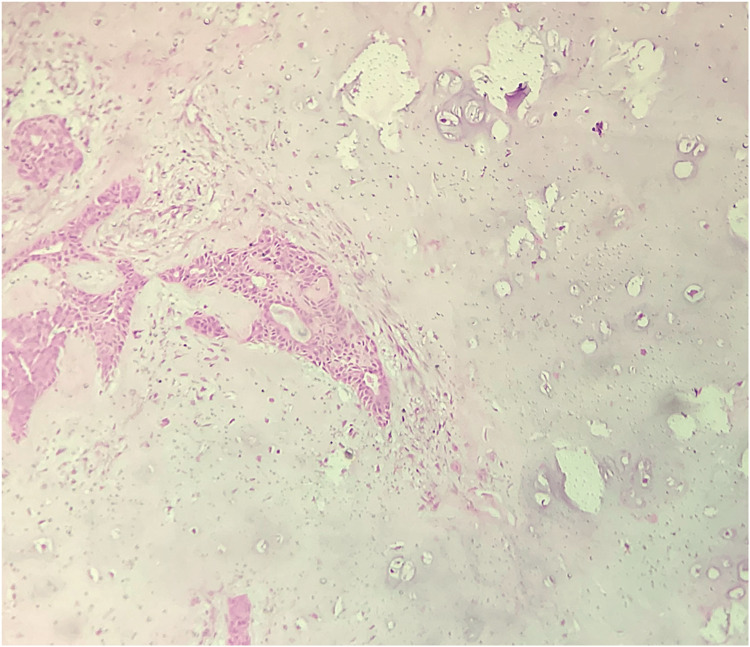
Biphasic tumor showing epithelial cells arranged in tubules and cartilage in myxoid background (chondroid syringoma), H&E 10X. H&E: hematoxylin and eosin stain, 10X: 10 times magnification.

Histopathology confirmed the diagnosis of chondroid syringoma as a biphasic tumor showing epithelial cells arranged in tubules and cartilage in a myxoid background (Figure 13).

**Figure 14:**
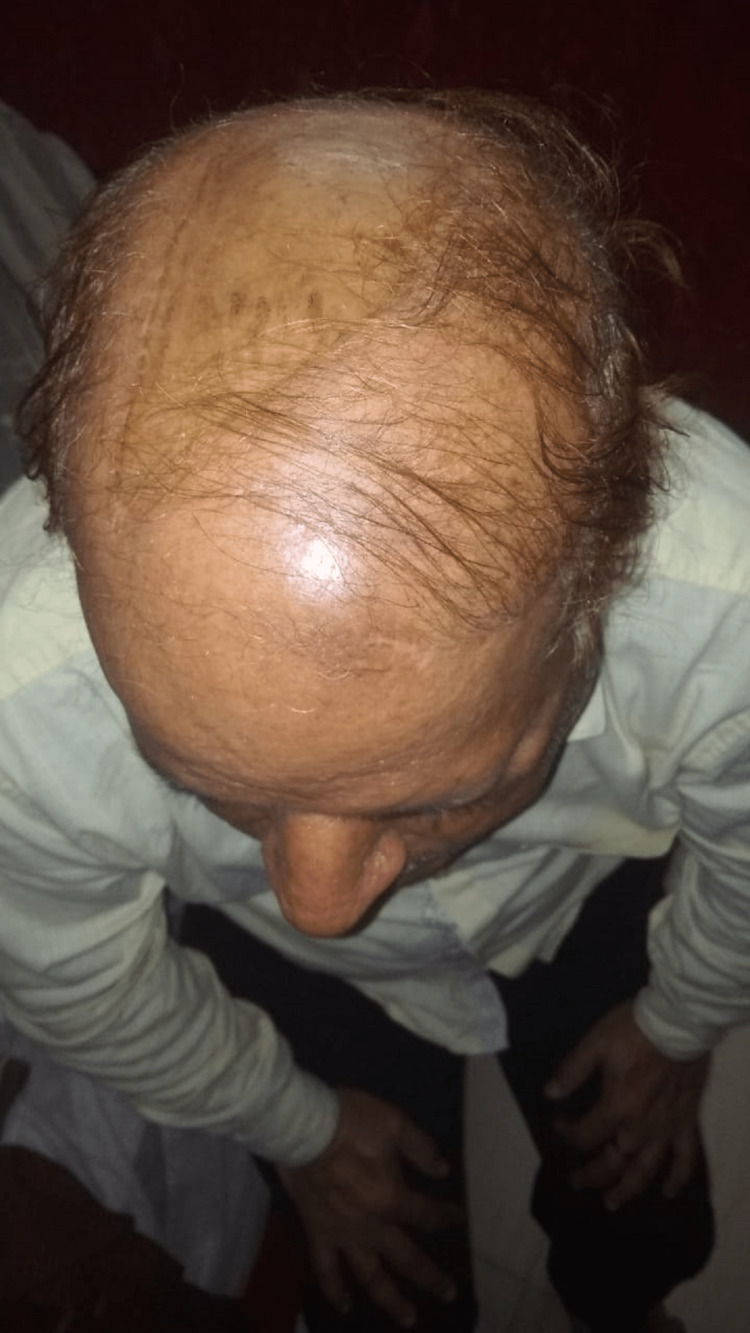
Five months post-surgery.

There was excellent healing and no sign of recurrence (Figure 14).

Case 4

**Figure 15:**
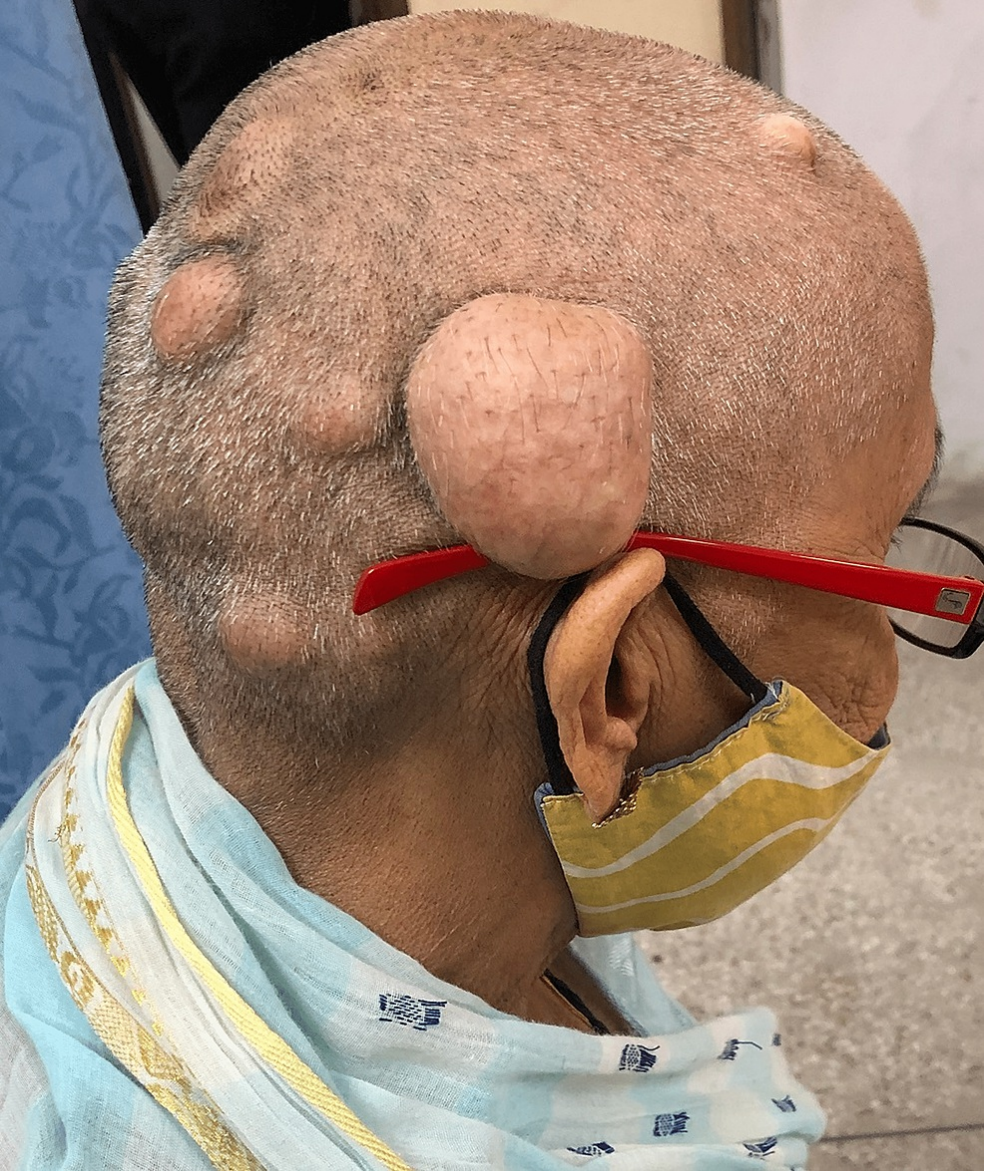
On presentation, multiple swellings on scalp.

A 75-year-old lady presented to the outpatient department with complaints of multiple swellings over the scalp for three years. The patient was apparently asymptomatic three years back when she noticed one swelling on the right side of the scalp approximately 2x2 cm in size. It was painless. The swelling increased in size progressively to the size of 5x5 cm. During this period, she also developed multiple small, similar swellings on the scalp. On examination, there was one large 5x5 cm swelling in the right temporal area and multiple small swellings ranging from 1x1 cm to 4x4 cm on the right half of the scalp. The swellings were spherical in shape, with normal overlying skin and hair. They were non-pulsatile and did not show cough impulse. The overlying temperature was normal, tenderness was absent, the surface was irregular, the edge was well-defined and the consistency was variable and moldable. They were fixed to the skin but free from underlying bone (Figure 15).

**Figure 16:**
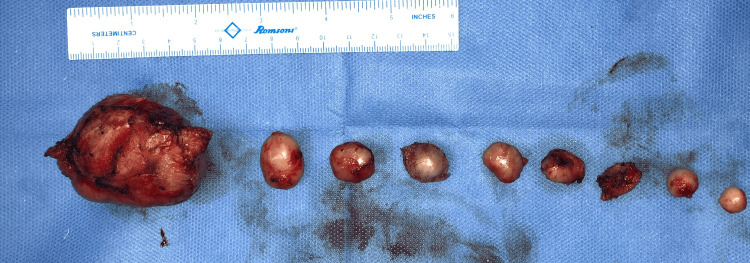
Excised cysts.

Non-contrast CT scan of the head was done which ruled out bony involvement. Excision of all the cysts was done under general anesthesia, and primary closure was achieved (Figure 16).

**Figure 17:**
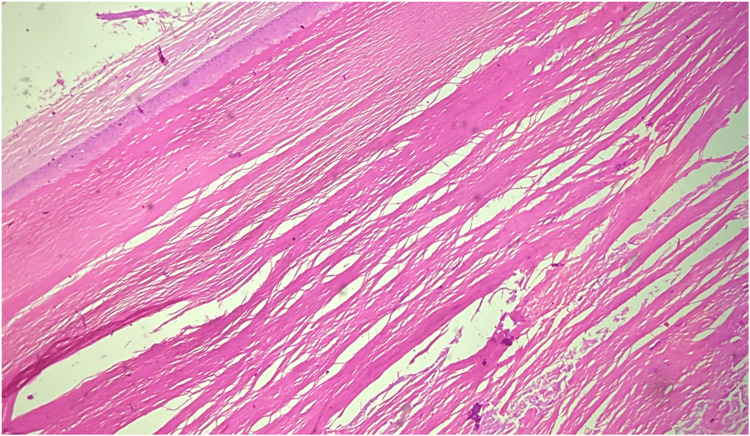
Trichilemmal cyst showing abrupt keratinization, H&E 10X. H&E: hematoxylin and eosin stain, 10X: 10 times magnification.

**Figure 18:**
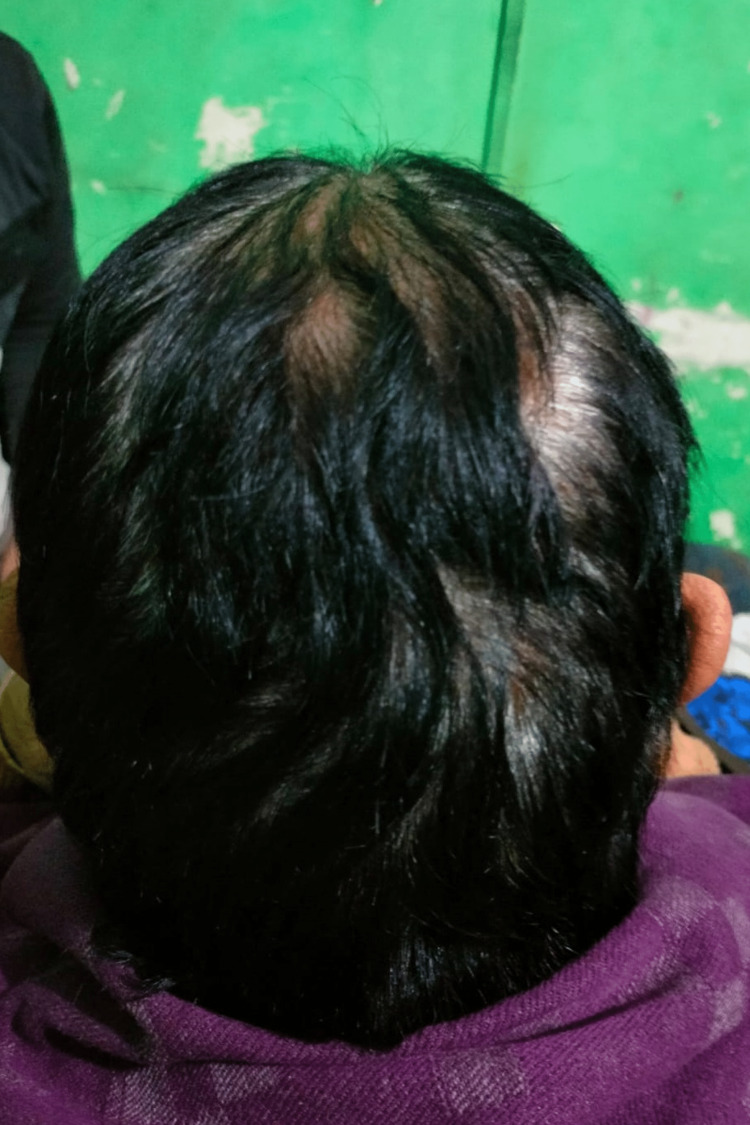
Four months post-surgery.

Histopathological examination revealed the diagnosis of a trichilemmal cyst (Figure 17). The post-operative course was unremarkable (Figure 18).

Case 5

**Figure 19:**
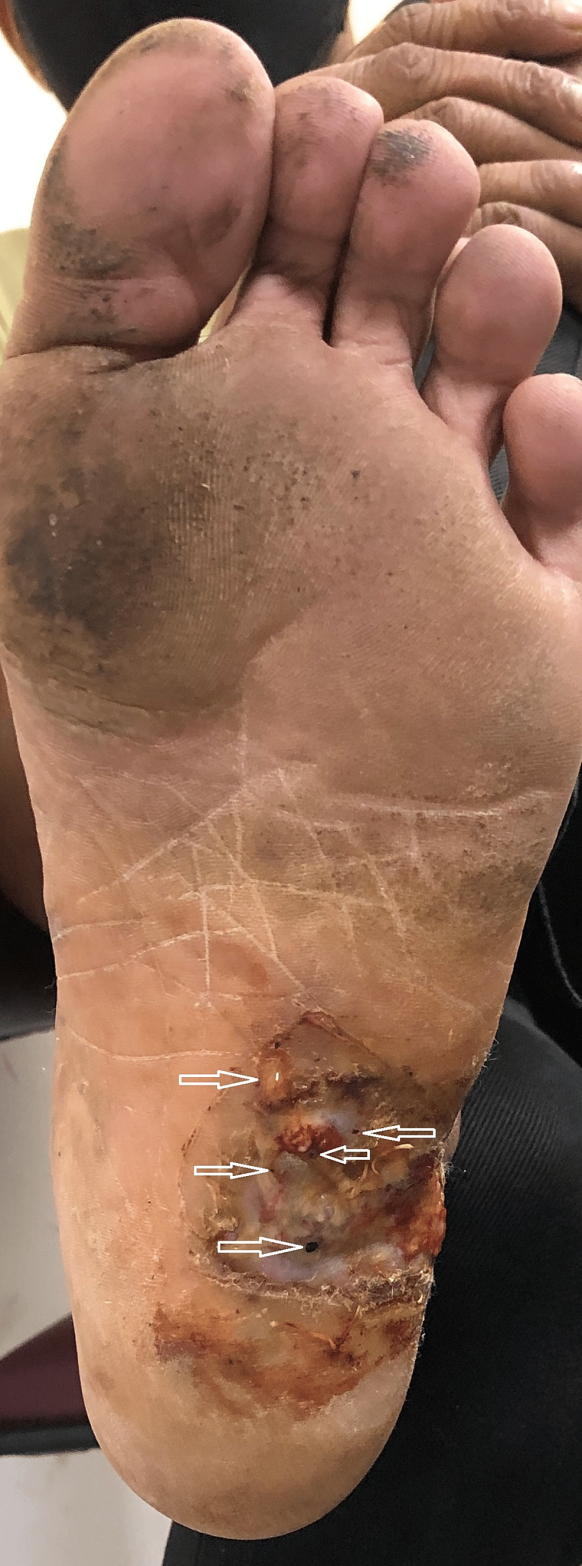
On presentation, the left sole showing ulcer with multiple discharging sinuses and yellow granules (white arrows).

A 28-year-old male presented to the outpatient department with complaints of a non-healing wound over the left foot for two years. The patient was apparently asymptomatic two years back when he noticed a wound on the left sole. It was about 1x1 cm in size and painless with pus discharge. The wound continued to increase in size and developed multiple openings which discharged pus and "grains" over the last two years. The patient was a farmer by occupation. On examination, there was an ulcer of 7x7 cm on the left foot on the sole just anterior to the heel and extending on the lateral aspect of the foot. Multiple sinus openings were present with yellow-colored granules (Figure 19). Ankle and toe movements were preserved.

**Figure 20:**
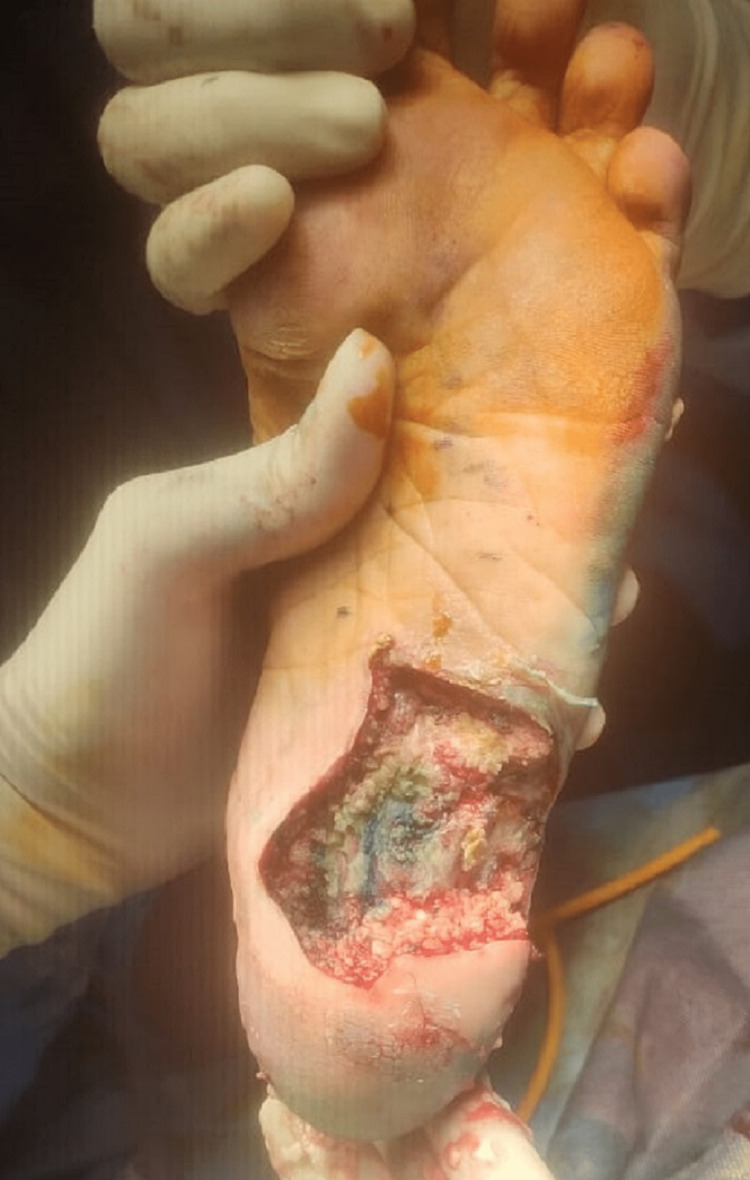
Post-debridement.

Microscopy of the grains revealed fungal hyphae. MRI of the left leg and foot showed multiple areas with dot in circle sign limited to myofascial planes and not involving the underlying bone which was suggestive of mycetoma of the foot without bone involvement. The patient was taken for debridement under anesthesia where all visibly involved tissue was debrided sparing the tendons and bones (Figure 20).

**Figure 21:**
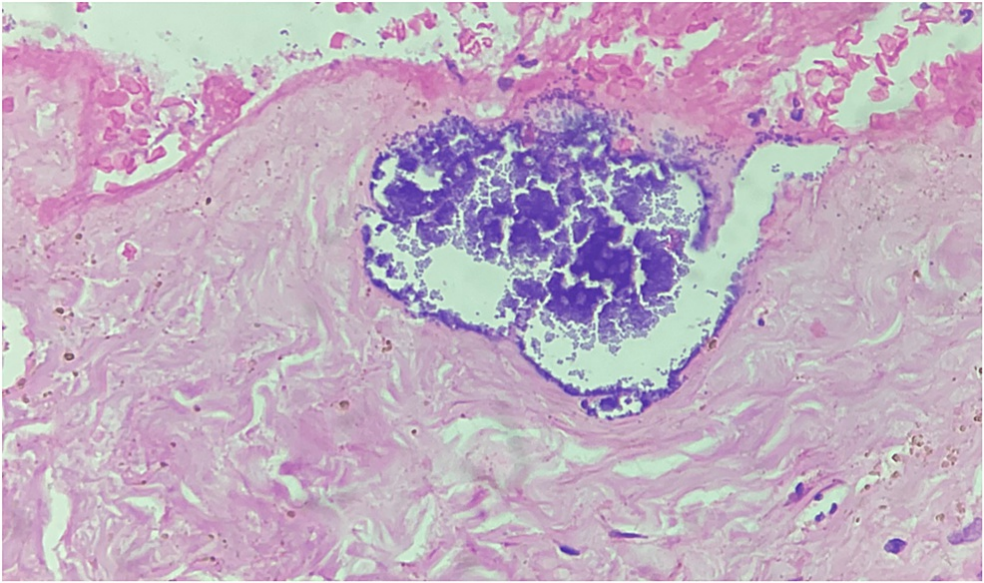
Mycetoma (Madura foot) showing granules in an inflammatory background, H&E 40X. H&E: hematoxylin and eosin stain, 40X: 40 times magnification.

The wound was kept open to heal by secondary intent. Microscopy of the excised specimen revealed actinomycosis. The patient was started on anti-bacterial and anti-fungal (Figure 21).

**Figure 22:**
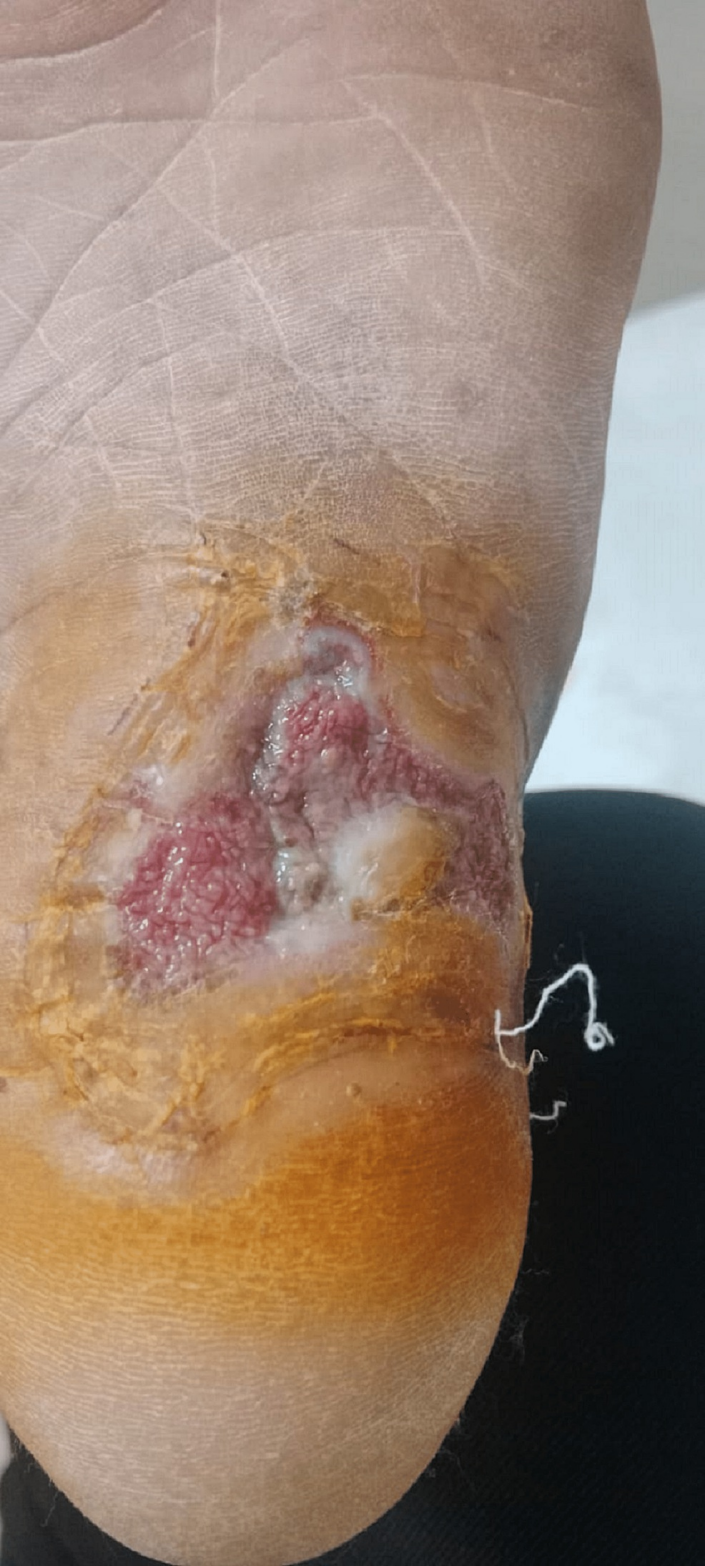
Three months post-debridement, healthy granulation.

The patient was followed up with regular dressing and showed good recovery with healthy granulation tissue at the wound site and no evidence of recurrence (Figure 22).

Case 6

**Figure 23:**
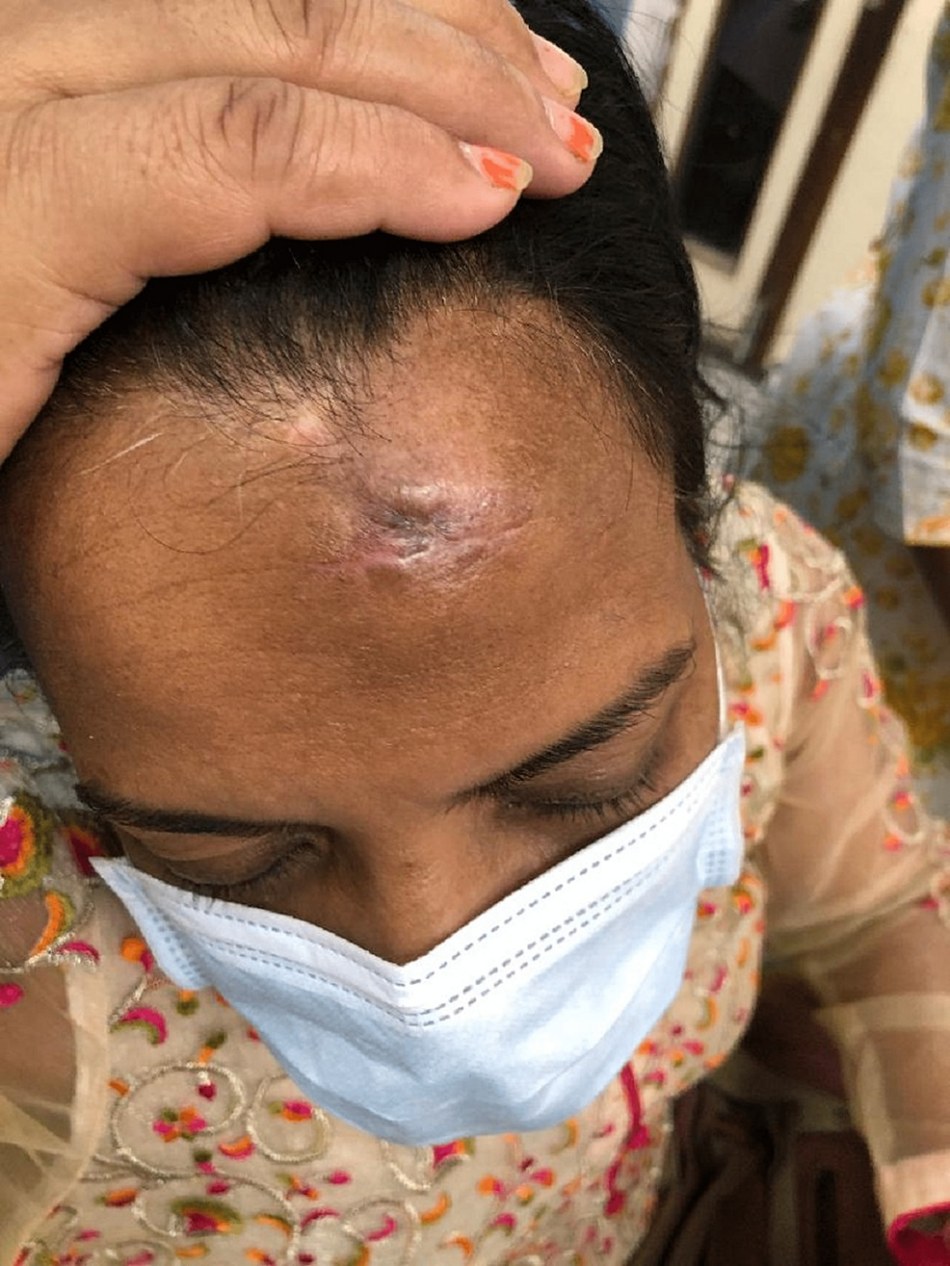
Button osteoma.

A 46-year-old female presented to the outpatient department with chief complaints of swelling over the forehead for 15 years. The lump was insidious in onset, approximately 2x2 cm in size, and had not progressed in size. There was no history suggesting trauma or infections. The swelling was painless and hard. The patient wished to get the swelling excised for cosmetic reasons. On examination, there was a 2x2 cm non-tender hard lump on the forehead which was fixed to the underlying bone and free from the overlying skin and subcutaneous tissue (Figure 23). A diagnosis of benign bony swelling was made. CT scan revealed a radiopaque lesion originating from the outer table of the skull. Excision of the swelling was done by open method using a chisel and mallet. Histopathology confirmed the diagnosis of osteoma.

## Discussion

Dermatofibrosarcoma protuberans

Dermatofibrosarcoma protuberans (DFSP) is a locally aggressive rare soft tissue tumor which has a high rate of local recurrence after surgical excision. DFSP has been described in the dermatology literature for more than a century. In 1925, Hoffman gave it its current name [1]. The cell of origin is thought to be a dermal fibroblast. According to a recent review of data from the National Cancer Institute's Surveillance, Epidemiology, and End Results (SEER) Program, DFSP has an incidence of 4.2 per million, which accounts for approximately 0.1% of all cancers [2]. DFSP is found in men and women at similar rates, with some large series indicating a slight male predominance [3]. In approximately 50% of cases, DFSP presents on the trunk. The next most common site is extremities, accounting for 20% to 35% of cases, with the head and neck affected less commonly, in 10% to 15% of cases [4]. DFSP typically presents as an asymptomatic skin-colored elevation that gradually enlarges over months to years, to become nodular. The tumors are usually adherent to the overlying skin, as a result of which the skin may undergo atrophy. DFSP is usually free to move over deeper structures, although long-standing tumors may be connected to underlying bone or fascia [5]. A patient with a history of a firm, slow-growing cutaneous nodule should be suspected of having DFSP. An incisional biopsy is required, and the final diagnosis is made using histopathological examinations with the assistance of immunohistochemical stains. Fine-needle aspiration should be avoided in general because it rarely yields a definitive diagnosis [6]. The classic microscopic appearance of DFSP on hematoxylin and eosin (H&E) staining is a proliferation of spindle cells in the dermis infiltrating into the subcutaneous fat. The cells in these proliferations are monotonous, with little pleomorphism and a low mitotic index. The spindle cells in the deep dermis are often arranged in a storiform or cartwheel pattern, and the infiltrating portion of the tumor is characterized by tentacle-like projections into the underlying fat, resulting in a honeycomb appearance [7]. It may be difficult to differentiate a dermatofibroma (DF) from DFSP on routine H&E staining. DFSP and DF have been distinguished using CD34 and factor XIIIa. Multiple studies have found that DFSP strongly expresses CD34, whereas the coagulation factor XIIIa is generally not expressed in DFSP but is expressed in DF strongly [8,9]. Radiological studies are not always necessary in cases of DFSP, but they can be helpful in determining the extent of the disease. Magnetic resonance imaging (MRI) has been found to be more accurate than Doppler ultrasonography in determining the extent of local tumor penetration [10]. Because the lymphatic spread of DFSP is uncommon, a physical examination with a focus on the regional lymph node basin is sufficient to screen for lymphatic metastases [11]. DFSP is aggressive locally but rarely metastasizes, with a metastasis rate estimated to be between 0.5% and 5% [12]. Dermatofibrosarcoma protuberans (DFSP) is a slow-growing tumor with a low metastatic potential but with significant subclinical extension and local destruction capacity. As a result, the first surgeon confronted with this tumor must attempt to cure the patient surgically such that it preserves healthy tissue while achieving the best oncological, functional and esthetic outcome. When available, Mohs micrographic surgery (MMS) is the preferred method; however, when MMS is not available, wide local excision (resection margins of at least 2 to 3 cm) with extensive pathologic assessment of margin status is recommended, and it is best to confirm tumor extirpation prior to any reconstruction [13]. National Comprehensive Cancer Network (NCCN) guidelines recommend wide margins of between 2 and 4 cm in the standard excision of DFSP [14].

Theoretically, MMS has a higher likelihood of providing complete surgical clearance because it is an asymmetric tumor with multiple eccentric microscopic projections which may span the dermis, subcutaneous tissue and fascia. [15]. A review of 58 patients with DFSP treated with MMS demonstrated that 70% of tumors extended at least 1 cm microscopically beyond the grossly visible lesion, 15% at least 3 cm microscopically and 5% at least 5 cm microscopically beyond the grossly visible lesion [15]. The recurrence rate of 49% was reported in a review of more than 100 cases in 1968. In several articles from the 1960s to 1980s, the recurrence rates for DFSP after local excision range from 10% to 60%. More recent studies advocating the use of local excision with wide surgical margins have documented a much lower rate of tumor recurrence. Imatinib became a potential treatment option after it was discovered that a translocation involving the tyrosine kinase platelet-derived growth factor beta (PDGFB) played a key role in the development of DFSP [16]. Imatinib is currently recommended by the NCCN guidelines in cases of DFSP recurrence after resection or in cases deemed unresectable [14].

Plexiform neurofibroma

Neurofibromatosis type 1 (NF1) is an autosomal dominant disorder caused by inherited or spontaneous mutations in the *NF1* gene, which encodes neurofibromin. The incidence is approximately 1:3,500 [17]. Plexiform neurofibromas are one of the most common causes of morbidity and cosmetic impairment in patients with NF1. A plexiform neurofibroma is characterized by cell proliferation of the nerve sheath cells that extends across the length of a nerve and involves multiple nerve fascicles. Plexiform neurofibromas can occur in up to 30% of NF1 cases. They occur most commonly in the craniomaxillofacial region [18]. They can be visible on the body's surface or may be internal, with no obvious superficial extension. Malignant progression is generally regarded as the leading cause of death, occurring in 2% to 16% of cases [18]. They transform into malignant peripheral nerve sheath tumors (MPNSTs). Plexiform neurofibroma is mostly a clinical diagnosis, and no radiological or tissue investigation is required as such. Abdomen and thorax contrast-enhanced CT scans may be performed to rule out deep-seated neurofibromas near vital organs. Surgical resection is the mainstay of management of plexiform neurofibroma where possible. The recurrence rates are high, up to 20% [19].

Chondroid syringoma

Chondroid syringoma (CS) is a rare skin appendageal tumor, with a reported incidence of 0.098% [20]. The cell of origin is thought to be from sweat glands. It usually affects middle-aged or older patients [21]. These lesions typically manifest as slow-growing, painless subcutaneous or intracutaneous nodules in the head and neck region [22]. The scrotum, eyelid, orbit, nose, foot, upper lip, forehead, axillary region, abdomen, penis, vulva and scalp are among the other predilection sites reported in the literature [23]. Histologically, CS is composed of an admixture of epithelial-myoepithelial structures arranged in cords and forming tubules within a chondromyxoid and fibrous stroma [24]. Hirsch and Helwig coined the term "chondroid syringoma" in 1961 due to the presence of sweat gland features in a cartilage-like stroma. Headington classified chondroid syringomas into apocrine and eccrine types based on histologic appearance [25,26]. Most cases have an unremarkable presentation as a benign mass, and thus, most diagnoses are made on microscopic examination post-excision. Occasionally, they may be diagnosed on fine-needle aspiration cytology. Malignant chondroid syringomas have been documented in literature which occur mainly in females and are more common in extremities [27]. Surgical excision remains the treatment of choice as it provides a specimen to rule out malignancy by microscopic examination. Other treatment options are electrodesiccation, dermabrasion and vaporization with argon or CO_2_ lasers.

Trichilemmal cyst

Trichilemmal cysts (TCs), also known as pilar cysts, are benign appendage lesions derived from the hair follicle's outer root sheath. Their incidence is 5%-10% and is more common in females [28]. Of all skin cysts, pilar cysts are the most common cysts. They occur mostly in areas with a high density of hair follicles, most commonly the scalp where they are often misdiagnosed as epidermal cysts (sebaceous cysts). Clinically, epidermal cysts and trichilemmal cysts may appear almost indistinguishable except for the fact that epidermal cysts usually have a punctum where as trichilemmal cysts lack a punctum. Microscopically trichilemmal cysts contain more keratin which is seen as homogenous eosinophilic content lined by stratified squamous epithelium. On the other hand, epidermal cysts contain lamellated keratin flakes lined by stratified squamous epithelium [29]. Trichilemmal cyst is a clinical diagnosis, and treatment is by excision of the cyst with its wall. A malignant variant of trichilemmal cyst has been described which is extremely rare and referred to as proliferating trichilemmal cysts (PTCs) or tumors [30].

Mycetoma

Mycetoma is a chronic suppurative infection affecting the skin, subcutaneous tissue and bones. It is endemic in India, Africa, South America and Mexico. It predominates in men with a 2-4:1 ratio. The average age of presentation ranges from 16 to 40 years [31]. Mycetomas are caused by various fungi and bacteria that live as saprophytes in soil or on plants. Aerobic actinomycetes from the genera *Nocardia*, *Streptomyces* and *Actinomadura* cause actinomycotic mycetoma, with *Nocardia brasiliensis*, *Actinomadura madurae*, *Actinomadura pelletieri* and *Streptomyces somaliensis* being the most common. Eumycotic mycetoma is associated with a variety of fungi, the most common being *Madurella mycetomatis* [32]. It is common in people who work on farms without proper foot protection. Individuals who frequently carry heavy objects on their shoulders or heads are at risk of infection in their hands, back and other exposed areas [33]. The microorganism multiplies in the subcutaneous tissue and spreads to neighboring tissues such as bone, skin and the sole or dorsum of the foot. Regardless of the organism involved, the clinical features are fairly consistent. A pathognomonic triad of a painless firm subcutaneous mass, multiple sinuses and purulent or seropurulent discharge containing grains is usually present [34]. Clinically, different species produce grains of different colors [35]. Because patients do not suffer much pain or disability, they delay their visits to hospitals and present at advanced stages. Only a microscopic examination of grain can reveal the causative organism. Histopathology and culture are usually not required. The characteristic sign on MRI is "dot in circle". Mycetoma treatment has proven to be difficult typically requiring antimicrobial agents and surgery in the form of debridement, and in extreme cases, amputation [35].

Button osteoma

Osteomas are benign bone tumors which can occur due to the proliferation of either cancellous or compact bone [36]. Facial and cranial bones are common locations for osteomas. Circumscribed small osteomas on the cranial vault are often referred to as button osteomas. Button osteomas can present to general surgeons, plastic surgeons or dermatologists. They usually present with asymptomatic small (rarely >1 cm in diameter) bony lumps on the parietal or frontal bone. They commonly occur from the second to the fifth decade of life and are more common in females [36]. Although the exact cause of button osteoma is unknown, it may be related to trauma, infection and developmental abnormalities. Plain radiography, ultrasound and computed tomography (CT) are all useful for diagnosis. Button osteoma is typically seen as radiopaque bone lesions that include the outer table of the skull. Management options include surgical removal of button osteoma, including ostectomy, curettage, endoscopic surgery and CO_2_ laser cauterization, usually performed for cosmetic reasons [36].

## Conclusions

We can thus conclude that a general surgeon with the knowledge of the latest literature and guidelines can effectively manage patients presenting with uncommon cutaneous disorders.

## References

[1] Hoffman E (1925). Ueber alas knollentribende fibrosarkam derhaut (dermatofibrosarcoma protuberans). Dermatol Z.

[2] Criscione VD, Weinstock MA (2007). Descriptive epidemiology of dermatofibrosarcoma protuberans in the United States, 1973 to 2002. J Am Acad Dermatol.

[3] Bowne WB, Antonescu CR, Leung DH (2000). Dermatofibrosarcoma protuberans: a clinicopathologic analysis of patients treated and followed at a single institution. Cancer.

[4] Chuang TY, Su WP, Muller SA (1990). Incidence of cutaneous T cell lymphoma and other rare skin cancers in a defined population. J Am Acad Dermatol.

[5] Gloster HM (1996). Dermatofibrosarcoma protuberans. J Am Acad Dermatol.

[6] Domanski HA, Gustafson P (2002). Cytologic features of primary, recurrent, and metastatic dermatofibrosarcoma protuberans. Cancer.

[7] Llombart B, Sanmartín O, López-Guerrero JA (2009). Dermatofibrosarcoma protuberans: clinical, pathological, and genetic (COL1A1-PDGFB ) study with therapeutic implications. Histopathology.

[8] Aiba S, Tabata N, Ishii H, Ootani H, Tagami H (1992). Dermatofibrosarcoma protuberans is a unique fibrohistiocytic tumour expressing CD34. Br J Dermatol.

[9] Silverman J, Tamsen A (1998). CD34 and factor XIIIa-positive microvascular dendritic cells and the family of fibrohistiocytic mesenchymal tumors. Am J Dermatopathol.

[10] Lee SJ, Mahoney MC, Shaughnessy E (2009). Dermatofibrosarcoma protuberans of the breast: imaging features and review of the literature. AJR Am J Roentgenol.

[11] Mavili ME, Gursu KG, Gokoz A (1994). Dermatofibrosarcoma with lymph node involvement. Ann Plast Surg.

[12] Lemm D, Mügge LO, Mentzel T, Höffken K (2009). Current treatment options in dermatofibrosarcoma protuberans. J Cancer Res Clin Oncol.

[13] Acosta AE, Vélez CS (2017). Dermatofibrosarcoma protuberans. Curr Treat Options Oncol.

[14] (2017). National Comprehensive Cancer Network: Dermatofibrosarcoma protuberans (Version 1.2018). https://www.nccn.org/professionals/physician_gls/pdf/dfsp.pdf.

[15] Ratner D, Thomas CO, Johnson TM (1997). Mohs micrographic surgery for the treatment of dermatofibrosarcoma protuberans: results of a multiinstitutional series with an analysis of the extent of microscopic spread. J Am Acad Dermatol.

[16] Shimizu A, O’Brien KP, Sjöblom T (1999). The dermatofibrosarcoma protuberans-associated collagen type I alpha1/platelet-derived growth factor (PDGF) B-chain fusion gene generates a transforming protein that is processed to functional PDGF-BB. Cancer Res.

[17] Friedman JM (2002). Neurofibromatosis 1: clinical manifestations and diagnostic criteria. J Child Neurol.

[18] Sabatini C, Milani D, Menni F, Tadini G, Esposito S (2015). Treatment of neurofibromatosis type 1. Curr Treat Options Neurol.

[19] Needle MN, Cnaan A, Dattilo J (1997). Prognostic signs in the surgical management of plexiform neurofibroma: the Children's Hospital of Philadelphia experience, 1974-1994. J Pediatr.

[20] Yavuzer R, Başterzi Y, Sari A, Bir F, Sezer C (2003). Chondroid syringoma: a diagnosis more frequent than expected. Dermatol Surg.

[21] Villalón G, Monteagudo C, Martin JM (2006). Chondroid syringoma: a clinical and histological review of eight cases. Actas Dermosifiliogr.

[22] Adlam DM, Wood GA (1986). The chondroid syringoma (mixed tumor of skin): report of a case in the upper lip. Oral Surg Oral Med Oral Pathol.

[23] Barnett MD, Wallack MK, Zuretti A, Mesia L, Emery RS, Berson AM (2000). Recurrent malignant chondroid syringoma foot. Am J Clin Oncol.

[24] Bates AW, Baithun SI (1998). Atypical mixed tumor of the skin: histologic, immunohistochemical, and ultrastructural features in three cases and a review of the criteria for malignancy. Am J Dermatopathol.

[25] Hirsch P, Helwig EB (1961). Chondroid syringoma: mixed tumor of skin, salivary gland type. Arch Dermatol.

[26] Headington JT (1961). Mixed tumors of skin: eccrine and apocrine types. Arch Dermatol.

[27] Mathiasen RA, Rasgon BM, Rumore G (2005). Malignant chondroid syringoma of the face: a first reported case. Otolaryngol Head Neck Surg.

[28] Chang SJ, Sims J, Murtagh FR, McCaffrey JC, Messina JL (2006). Proliferating trichilemmal cysts of the scalp on CT. AJNR Am J Neuroradiol.

[29] Weedon D (2002). Skin Pathology, 2nd Ed.

[30] Ramaswamy AS, Manjunatha HK, Sunilkumar B, Arunkumar SP (2013). Morphological spectrum of pilar cysts. N Am J Med Sci.

[31] Arenas R (2015). Micetoma. Dermatologıa: Atlas, Diagnostico y  Tratamiento, 6a Edn.

[32] Relhan V, Mahajan K, Agarwal P, Garg VK (2017). Mycetoma: an update. Indian J Dermatol.

[33] Lichon V, Khachemoune A (2006). Mycetoma: a review. Am J Clin Dermatol.

[34] Mahgoub ES (1976). Medical management of mycetoma. Bull World Health Organ.

[35] Magana M (1984). Mycetoma. Int J Dermatol.

[36] Haddad FS, Haddad GF, Zaatari G (1997). Cranial osteomas: their classification and management report on a giant osteoma and review of the literature. Surg Neurol.

